# Mitochondrial dysfunction induced in human hepatic HepG2 cells exposed to the fungicide kresoxim-methyl and to a mixture kresoxim-methyl/boscalid

**DOI:** 10.1080/13510002.2024.2424677

**Published:** 2024-11-14

**Authors:** Yasmine Vandensande, Mélina Carbone, Barbara Mathieu, Bernard Gallez

**Affiliations:** Biomedical Magnetic Resonance Research Group, Louvain Drug Research Institute, Brussels, Belgium

**Keywords:** Fungicides, mitochondrial function, Electron Paramagnetic Resonance (EPR), cocktail effect, strobilurin, SDHI

## Abstract

The fungicides strobilurins and succinate dehydrogenase inhibitors (SDHIs) are blockers of the electron transport chain (ETC) in fungi. Here, we show that the exposure for 24 h to kresoxym-methyl, a fungicide from the class of strobilurins, alters the mitochondrial respiration in human HepG2 hepatocytes. In addition, we demonstrate an increase in production of mitochondrial superoxide radical anion, a reduction in ATP level, a decrease in the ratio reduced/oxidized glutathione and a decrease in cell viability (assessed by the LDH assay, Presto Blue assay, and Crystal Violet assay). As kresoxym-methyl is associated to boscalid (SDHI) in commercial formulations, we analyzed a potential exacerbation of the induced mitochondrial dysfunction for this combination. For the highest dose at which kresoxym-methyl (5 µM) and boscalid (0.5 µM) did not induce changes in mitochondrial function when used separately, in contrast, when both fungicides were used in combination at the same concentration, we observed a significant alteration of the mitochondrial function of hepatocytes: there was a decrease in oxygen consumption rate, in the ATP level. In addition, the level of mitochondrial superoxide radical anion was increased leading to a decrease in the ratio reduced/oxidized glutathione, and an increase in viability.

## GRAPHICAL ABSTRACT

**Figure:**
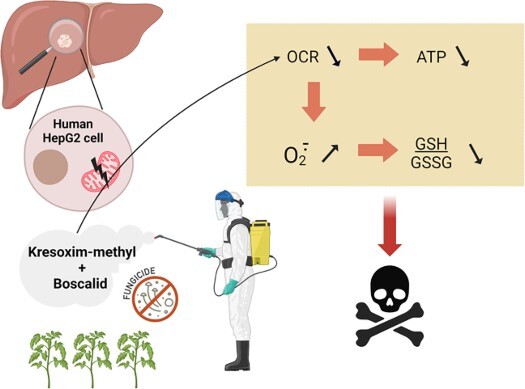

## Introduction

Strobilurins form a class of fungicidal compounds that were initially isolated from Strobilurus tenacellus commonly known as the pinecone cap, a fungus that grows on the fallen cones of pine and spruce trees [1]. Synthetic analogs similar to strobilurin A (isolated from S. tenacellus) were developed to improve fungicidal activity and environmental stability [2]. From these efforts, emerged kresoxim-methyl (methyl(E)-methoxyimino-2-[2-(o-tolyloxymethyl)phenyl] acetate, initially referred as BAS 490 F), an active fungicide that specifically inhibits respiration by binding to the mitochondrial cytochrome bc1 complex (complex III) of the electron transport chain [2,3]. Kresoxim-methyl inhibits the growth of mildews, blast, and sheath blight and is largely used worldwide under different commercial brand names (Ayaan®, BAS 490®, Cygnus®, Ergon®, Kriman®, Sarthak®, …). Strobilurins were initially considered as relatively safe for ecosystems. However, in the past few years, a series of studies reported adverse effects on soils organisms, aquatic organisms and mammals (for recent reviews, see [4,5]). Of note, most reports on strobilurins analyzed the effects of azoxystrobin, fluoxastrobin, trifloxystrobin and pyraclostrobin. Kresoxym-methyl has been less studied, but this compound has been shown to change the activity of antioxidant enzymes in grass carp juveniles [6] and in algae [7], to induce mitochondrial dysfunction, metabolic disorders and alteration in the development of zebrafish [8–13], to alter redox balance in a monkey renal cell line [14], to cause neurotoxicity in primary cultured mouse cortical neurons [15], and to alter cellular homeostasis of murine neuroblastoma [16]. Intriguingly, studies reporting the impact of the exposure to strobilurins on human cells are scarce. It was found that trifloxystrobin induces TRAIL-mediated apoptosis and mitochondrial damage in keratinocytes from human skin [17,18], that azoxystrobin and trifloxystrobin inhibit mitochondrial oxidative respiration and alter the abundance of several lipids in neuronal cells [19], and that several strobilurins were toxic for the human hepatocytes HepG2 [20–22].

Because the target of strobilurins is the complex III of the ETC and because mitochondrial perturbations can lead to major effects on metabolism, energy store and production of reactive oxygen species (ROS), it is worth to investigate the consequences of the exposure of human cells to strobilurins. Here, because kresoxym-methyl has been less studied than other strobilurins, we focused on the exposure of human hepatic HepG2 cells exposed to this compound. Kresoxym-methyl is rather lipophilic (logP = 3.4), rapidly absorbed (0.5–1 h after oral administration) with a bioavailability of 63% [23] and a half-life of 19–22 h, with the highest concentrations of compound found in the gut, liver and kidneys after oral administration [24]. AOEL (Acceptable Operator Exposure Level) and ADI (Acceptable Daily Intake) for this compound are 0.9 mg/kg bw/day and 0.3 mg/kg bw/day, respectively. Considering an oral absorption of 63%, for a person of 70 kg body weight with a volume of blood of 5 L, the peak of concentration in the blood would achieve a value around 8 µM or 24 µM after a single exposure dose close to the ADI or the AOEL, respectively. Therefore, our first investigations were carried out in this relevant range of concentration. The study design was similar to our recent study performed on pyraclostrobin, another strobilurin [21]. We first screened a dose-dependent effect on the oxygen consumption rate (OCR) to define the lowest concentration that induced a significant effect on HepG2 cells. Because an alteration of the mitochondrial function may increase the leakage of free electrons and an excessive production of the mitochondrial superoxide radical anion, we evaluated its production by Electron Paramagnetic Resonance (EPR) spectroscopy. We also explored the consequences of the induced mitochondrial dysfunction in terms of ATP level (marker of energy store), the ratio between reduced and oxidized glutathione (redox state marker of oxidative stress), and cytotoxicity. In our previous study, we highlighted a potential cocktail toxic effect for the association strobilurin/SDHI (succinate dehydrogenase inhibitor) using pyraclostrobin/boscalid [21]. Therefore, here, we also investigated a potential similar exacerbation of toxicity for the mixture kresoxym-methyl/boscalid as both compounds are associated in several commercial preparations (Camro®, Castra®, Collis®, …).

## Methods and materials

### Reagents and cell culture

Kresoxym-methyl PESTANAL (CAS number: 143390-89-0) and Boscalid PESTANAL (CAS number:188425-85-6) were purchased from Supelco (Sigma Aldrich, Hoeilaert, Belgium) and dissolved in DMSO (Sigma Aldrich, Hoeilaert, Belgium). HepG2 cells were acquired from the American Type Culture Collection (ATCC) (Manassas, VA, U.S.A.) and cultured in MEM no nucleosides medium (Thermo Fischer, Merelbeke, Belgium) with 10% of heat-inactivated fetal bovine serum (FBS) (Sigma Aldrich). Cells were maintained at 37°C in humidified atmosphere with 5% CO_2_.

### Oxygen consumption rate

EPR spectroscopy was used to measure variations in oxygen levels in cell media over time [25, 26]. The principle is based on the analysis of the EPR linewidth of an oxygen-sensing probe, ^15^N-PDT (4-oxo-2,2,6,6-tetramethylpiperidine-d_16_-^15^N-1-oxyl) (CDN Isotopes, Pointe-Claire, QC, Canada). As the linewidth of ^15^N-PDT has been calibrated as a function of the % oxygen present in the sample, it allows the monitoring of OCR in a given preparation of cells. The full detail procedure for measuring OCR by EPR has been described elsewhere [27]. 24 h before the experiment, the cells were treated with fungicides (kresoxym-methyl and/or boscalid) or the appropriate control (DMSO, final concentration 0.1% v/v). The cells were collected to form a stock solution of 5 × 10^6^ cells/mL of culture medium. A mixture containing 60 µL of cell suspension, 40 µL of Dextran solution (20% m/v, from leuconostoc mesenteroides, MW 60-76,000, Sigma Aldrich, Hoeilaert, Belgium), and 4 µL of ^15^N-PDT (2 mM) was placed into a hematocrit capillary sealed with gum. The sealed capillary was inserted into a quartz tube and put into the EPR cavity heated at 37°C with continuous nitrogen flow. The OCR of cells was measured using a Bruker EMX-Plus spectrometer operating in X-band (9.85 GHz). The experimental parameters were: microwave power, 2.518 mW; modulation amplitude, 5 µT; modulation frequency, 100 kHz; center field, 335 mT; sweep time, 15 s; and sweep width, 1.5 mT. The measurement started 2 min after having mixed ^15^N-PDT with the cells. EPR spectra were recorded each minute to monitor evolution of oxygen concentration as a function of time.

### Mitochondrial superoxide radical anion measurement with EPR

The method is based on the transformation of a cyclic hydroxylamine Mito-TEMPO-H (2-(2,2,6,6-Tetramethylpiperidin-1-oxyl-4-ylamino)−2-oxoethyl triphenylphosphonium chloride) (Enzo Lifescience, Antwerpen, Belgium) into a paramagnetic nitroxide in the presence of reactive species [28,29]. This compound accumulates in mitochondria due to its tryphenylphosphonium (TPP^+^) group. The full detailed procedure has been described elsewhere [27]. 24 h before the experiment, the cells were treated with fungicides (kresoxym-methyl and/or boscalid) or the appropriate control (DMSO, final concentration 0.1% v/v). The cells were collected to form a stock solution of 15 × 10^6^ cells/mL of culture medium. A mixture containing 37 µL of cell suspension, 0.50 µL of DTPA (100 mM), 5 µL of PBS ((1x) – pH 7.4), and 7.5 µL of Mito-TEMPO-H (1 mM) was placed into a gas-permeable polytetrafluoroethylene tube (inside diameter 0.025 in., wall thickness 0.002 in.) inserted into an open quartz tube. The quartz tube was inserted in the EPR cavity and heated at 37°C with continuous air flow. The formation of nitroxides can be actually due to the presence of superoxide radical anion, but also reaction with other ROS species such as peroxynitrite, and derived nitrogen species. Therefore, to evaluate the specific contribution of the superoxide radical anion to the oxidation of the probe, control experiments were carried out with PEG-SOD_2_ as a scavenger of the superoxide radical anion [30]. Experiments were done using the same conditions by adding 2.5 µL of PEG-SOD_2_ (4000 U/mL) incubated for 15 min before adding the Mito-TEMPO-H probe, allowing the cellular uptake of the enzyme and the intracellular scavenging of superoxide. The superoxide radical anion production was monitored using the same spectrometer described for the OCR measurements. The experimental parameters were: microwave power, 20 mW; 100 kHz; modulation amplitude, modulation frequency, 0.1 mT; center field, 336.5 mT; sweep time, 30.5 s; and sweep width,1.5 mT. The measurement started 2 min after mixing the probe with the cells and repeated with a time delay of 40 s between each point. Double integration of the central peak was used to assess the amount of nitroxide produced.

### Cell viability

Three assays were used to assess the cytotoxicity: the Presto Blue assay that reflects cytotoxicity through altered metabolic mitochondrial activity, the Crystal Violet assay that stains the nuclear DNA and the proteins of adherent cells, and lactate dehydrogenase (LDH) release assay that quantifies the release of this cytosolic enzyme reflecting cell plasma membrane damage due to cytotoxicity. The protocol we used for the Presto Blue and Crystal Violet assays were described previously [21]. For all three assays, the cells were seeded in a 96-well plate at the density of 10,000 cells/well two days before the experiment. The cells were treated with fungicides (kresoxym-methyl and/or boscalid) or the appropriate control (DMSO, final concentration 0.1% v/v) for 24 h. For the Presto Blue assay, mix of cell medium with 10% (v/v) PrestoBlue cell viability reagent (ThermoFisher) was added to each well and incubated for 2 h at 37°C, protected from the light. Fluorescence was measured at 560 and 590 nm on a SpectraMaxE2 plate reader (Molecular Devices). For the Crystal Violet assay, the cells were washed twice with PBS and 50 µL of Crystal Violet solution (1%) (CAS number: 548-62-9) (Sigma-Aldrich, Hoeilaert, Belgium) and incubated for 20 min at room temperature on an orbital plate (20 oscillations/min). Then, the cells were washed with PBS to eliminate the excess of the Crystal Violet solution and the remaining dead cells. The stain dried for 2 h and was resuspended in methanol. The absorbance was measured at 570 nm. For the LDH assay, the CyQUANTTM LDH Cytotoxicity Assay Kit (Invitrogen, Thermo Fisher Scientific, Dilbeek, Belgium) was used to perform the LDH assay. LDH was quantified via a coupled enzymatic reaction: LDH catalyzes the conversion of lactate to pyruvate via the reduction of NAD^+^ to NADH. In the presence of diaphorase, the NADH formed in the previous reaction enables the reduction of tetrazolium to a red formazan product, which can be measured spectrophotometrically. Three parameters were measured: maximum LDH release, spontaneous LDH release and LDH release in cells exposed to fungicides. These parameters were quantified following the manufacturer’s instructions. Absorbance was measured with a SpectraMaxE2 plate reader (Molecular Devices) at the wavelength of 490 and 680 nm. To determine LDH activity, the absorbance measured at 680 nm, equivalent to the background, was subtracted from the absorbance measured at 490 nm. All results are presented as a percentage of the control condition (DMSO).

### Ratio oxidized / reduced glutathione

Intracellular glutathione levels were assessed using a protocol previously described [21]. The Glutathione colorimetric detection kit (Invitrogen, Waltham, MA, U.S.A., Thermo Fischer) was used. Cells were treated for 24 h with fungicides (kresoxym-methyl and/or boscalid) or the DMSO (control, final concentration 0.1% v/v). The total glutathione (GSHtot) and the oxidized form (GSSG) were quantified according the manufacturer’s instructions. The absorbance was measured with a SpectraMaxE2 plate reader (Molecular Devices) at the wavelength of 405 nm. The reduced form (GSH) was deduced via the subtraction of GSHtot from the GSSG concentration. The proteins were quantified for each condition using a BCA Protein Assay kit (PierceTM, Thermo Fisher). The concentration of intracellular glutathione was normalized by mg of protein. L-Buthionine sulfoximine (L-BSO, 25 µM, Sigma-Aldrich, Hoeilaert, Belgium), a glutathione synthase inhibitor, was used as a positive control.

### Intracellular ATP

Celltiter-Glo® Luminescent cell viability assay (Promega, Madison, WI, U.S.A.) was used to quantify the intracellular ATP. The cells were seeded in a 96-well plate at the density of 7,500 cells/well 48 h prior to quantification. Cells were treated for 24 h with fungicides (kresoxym-methyl and/or boscalid) or DMSO (control). Cells were washed twice with PBS (pH 7.4, Thermo Scientific, Waltham, MA, U.S.A.), and the Celltiter-Glo® Reagent mixed in PBS was added following the manufacturer’s instructions. The luminescence was measured after 5 min of incubation using a SpectraMaxE2 plate reader (Molecular Devices, San Jose, CA, U.S.A.). The quantity of ATP was assessed from a calibration curve of ATP disodium (Sigma-Aldrich, Hoeilaert, Belgium) from 10 nM to 1 µM. For each condition, the protein quantification was performed using a BCA Protein Assay Kit (PierceTM, ThermoFisher). The concentration of intracellular ATP was normalized by mg of protein and presented as a percentage of the control condition (DMSO). Oligomycin (0.1 µg/mL m/v, Sigma-Aldrich, Hoeilaert, Belgium), an ATP synthase inhibitor, was used as a positive control.

### Statistical analysis

The statistical analysis was performed on GraphPad Prism 9.5 Software. For each experiment, the normality of the data was verified with the Shapiro–Wilk test. The test and number of replicates are provided in each figure. The data shown are mean ± SEM.

## Results

### Kresoxim-methyl induces a mitochondrial dysfunction in human hepatocytes

**Figure 1.**
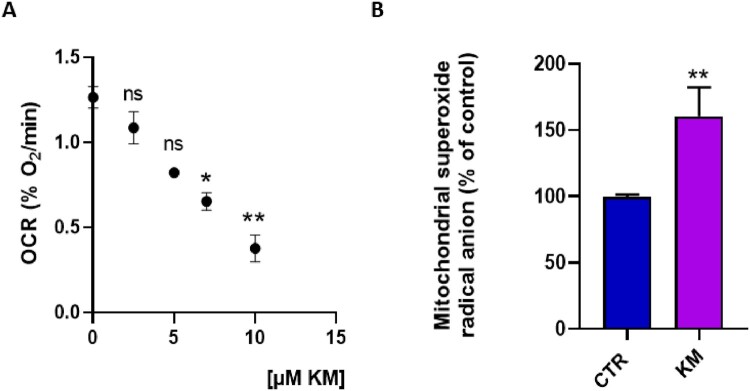
**(A)** Dose-effect relationship of kresoxim-methyl on the oxygen consumption rate established by EPR oximetry, N = 3, Anova One-way, Dunnett’s multiple comparison test. Impact of 24 h exposure to kresoxim-methyl 7 μM on HepG2 cells. CTR = control, KM = kresoxim-methyl 7 µM **(B)** level of mitochondrial superoxide radical anion, N = 3, T-test. Bars represent mean ± SEM. (*): *p* < 0.05, (**): *p* < 0.01, ns: not significant. *p* values are compared to control.

To study mitochondrial function following exposure to the fungicide kresoxym-methyl, we first measured the OCR by EPR oximetry in HepG2 cells. The dose-effect relationship study revealed a significant decrease in OCR for concentrations equal to or greater than 7 µM of kresoxim-methyl, compared with untreated cells (Figure 1A). The concentration of 7 µM was therefore selected to assess the impact of kresoxim-methyl on various parameters linked to a potential mitochondrial dysfunction. After 24 h exposure to kresoxim-methyl, we observed a significant increase in the level of mitochondrial superoxide radical anion in HepG2 cells (Figure 1B).

**Figure 2.**
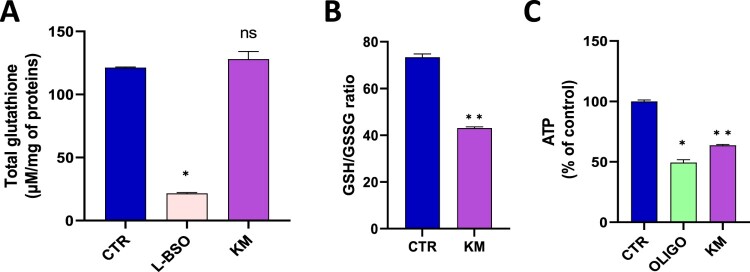
**(A)** level of total glutathione (reduced GSH and oxidized GSSG forms), N = 3, Anova One-way, Dunnett’s multiple comparison test. CTR = control, OLIGO = oligomycin, BSO = buthionine-sulfoximine, KM = kresoxim-methyl 7 µM **(B)** ratio between the levels of reduced GSH and oxidized GSSG forms, N = 6, T-test; **(C)** Level of ATP, N = 3, T-test. Bars represent mean ± SEM. (*): *p* < 0.05, (**): *p* < 0.01, ns: not significant. *p* values are compared to control.

Contrarily to BSO, an irreversible inhibitor of γ-glutamylcysteine synthetase used as a positive control, the exposure to kresoxim-methyl did not significantly alter the level of total glutathione (Figure 2A). In contrast, the ratio GSH/GSSG was significantly reduced after 24 h exposure to 7 µM of kresoxim-methyl, suggesting a consumption of the reduced GSH by the overproduction of ROS (Figure 2B). The level of ATP was also significantly decreased showing an alteration in the energetic metabolism (Figure 2C).

**Figure 3.**
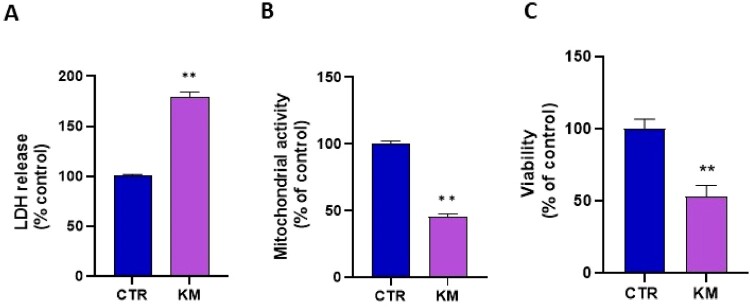
**(A)** cytotoxicity assessed by LDH assay, N = 3, T-test. CTR = control, KM = kresoxim-methyl 7 µM (**B**) mitochondrial activity assessed by the Presto Blue assay, N = 3, T-test; (**C**) viability assessed by the Crystal Violet assay, N = 3, T-test. Bars represent mean ± SEM. (*): *p *< 0.05, (**): *p *< 0.01. *p* values are compared to control.

Finally, we studied the cytotoxicity by three different assays that provided consistent results suggesting an alteration of cell viability after 24 h exposure to 7 µM of kresoxim-methyl: we observed a significant increase in LDH release (Figure 3A), decrease in mitochondrial activity (Presto Blue assay) (Figure 3B) and decrease in Crystal Violet staining (Figure 3C). Of note, exposure during 2 h or 8 h did not lead to a significant cytotoxicity (Supplementary Figure 1).

### The mixture of kresoxim-methyl and boscalid potentiates a mitochondrial dysfunction in human hepatocytes

**Figure 4.**
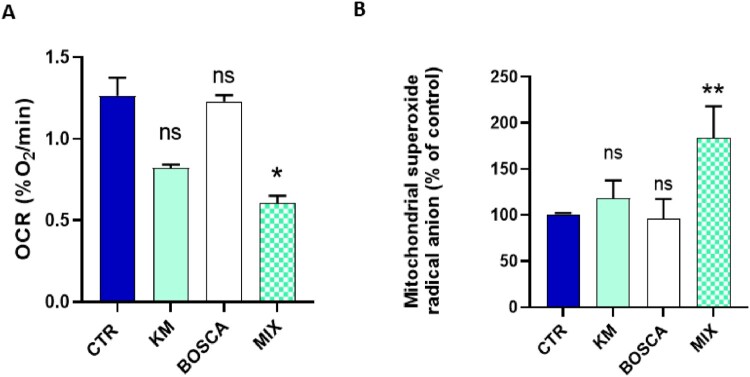
Impact of 24 h exposure to a kresoxim-methyl 5 μM/boscalid 0.5 µM mixture on HepG2cells. CTR = control, OLIGO = oligomycin, BSO = buthionine-sulfoximine, KM = kresoxim-methyl 5 µM, BOSCA = boscalid 0.5 µM, MIX = kresoxim-methyl 5 μM/boscalid 0.5 µM mixture **(A)** oxygen consumption rate, N = 3, Anova One-way, Dunnett’s multiple comparison test; **(B)** level of mitochondrial superoxide radical anion, N = 3, Anova One-way, Dunnett’s multiple comparison test. Bars represent mean ± SEM. (*): *p* < 0.05, (**): *p* < 0.01, ns: not significant. *p* values are compared to control.

**Figure 5.**
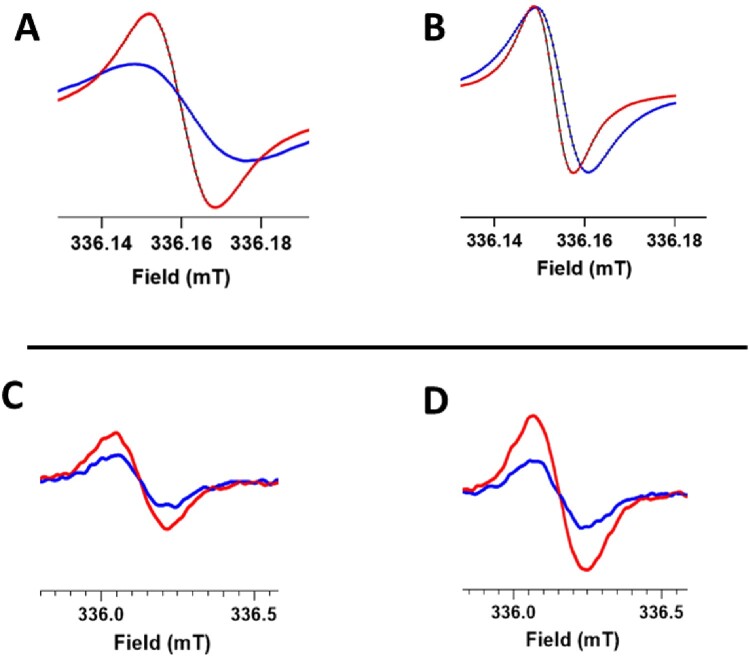
Representative EPR spectra showing the impact of 24 h exposure to a kresoxim-methyl 5 μM/boscalid 0.5 µM mixture on HepG2cells. Top row: OCR experiment where the oxygen concentration is derived from the evolution over time of the EPR linewidth of ^15^N-PDT (high-field peak). Blue spectra were acquired 2 min after mixing the oxygen sensor with the cell suspension, red spectra were acquired 10 min later. Note the large evolution of EPR linewidth for control cells (A) while the change in EPR linewidth was much smaller for cells that have been exposed to the mixture of fungicides (B). Bottom row: evolution over time of mitochondrial ROS production detected by the transformation of mitoTEMPO-H into nitroxide (center field peak). Blue spectra were acquired 2 min after mixing mitoTEMPO-H with the cell suspension, red spectra were acquired 10 min later. Note the small increase in mitochondrial ROS production in control cells (C) and the larger increase in mitochondrial ROS production for cells previously exposed to the mixture of fungicides.

To fight against resistance, kresoxim-methyl is often mixed with boscalid (SDHI) in various commercial preparations. To evaluate a potential exacerbation of mitochondrial dysfunction for this mixture of two fungicides, we selected, for each compound, the higher concentrations that did not induce a mitochondrial dysfunction when used individually. While no significant effect was observed at the concentration of 5 µM for kresoxim-methyl and 0.5 µM for boscalid on OCR (Figure 4A) and on mitochondrial superoxide radical anion production (Figure 4B), we observed that combined exposure to both fungicides at the same concentration induced a significant decrease in OCR (Figure 4A) and a significant increase in the mitochondrial superoxide radical anion production (Figure 4B). In Figure 5, we present representative EPR spectra showing the evolution of OCR and mitochondrial ROS over time in control cells and cells exposed to a mixture kresoxym-methyl/boscalid.

**Figure 6.**
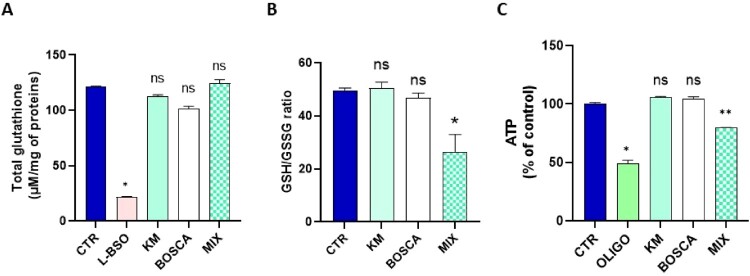
(**A**) level of total glutathione (reduced GSH and oxidized GSSG forms), N = 3, Anova One-way, Dunnett’s multiple comparison test. CTR = control, OLIGO = oligomycin, BSO = buthionine-sulfoximine, KM = kresoxim-methyl 5 µM, BOSCA = boscalid 0.5 µM, MIX = kresoxim-methyl 5 μM/boscalid 0.5 µM mixture (**B**) ratio between the levels of reduced GSH and oxidized GSSG forms, N = 3, Anova One-way, Dunnett’s multiple comparison test; (**C**) level of ATP, N = 3, Anova One-way, Dunnett’s multiple comparison test. Bars represent mean ± SEM. (*): *p *< 0.05, (**): *p *< 0.01, ns: not significant. *p* values are compared to control.

While the exposure to the mixture did not alter the level of total glutathione (Figure 6A), we observed a significant decrease in the ratio GSH/GSSG for the mixture (Figure 6B). We also observed that the level of ATP was significantly decreased in HepG2 cell after the exposure to the mixture (Figure 6C).

**Figure 7.**
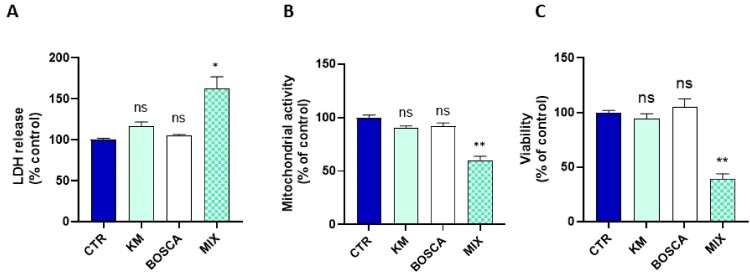
(A) Cytotoxicity assessed by LDH assay, N = 3, Anova One-way, Dunnett’s multiple comparison test; CTR = control, KM = kresoxim-methyl 5 µM, BOSCA = boscalid 0.5 µM, MIX = kresoxim-methyl 5 μM/boscalid 0.5 µM mixture (B) mitochondrial activity assessed by the Presto Blue assay, N = 3, Anova One-way, Dunnett’s multiple comparison test; (C) viability assessed by the Crystal Violet assay, N = 3, Anova One-way, Dunnett’s multiple comparison test. Bars represent mean ± SEM. (*): *p* < 0.05, (**): *p* < 0.01, ns: not significant. *p* values are compared to control.

The cytotoxicity was significantly increased after 24 h exposure to the mixture (Figure 7A). Finally, the three viability assays revealed a significant cytotoxicity after the combined exposure to both fungicides for 24 h (Figures 7 A-B-C). Results for shorter exposure are presented in Supplementary Figure 1.

## Discussion

The two main results of the present study are: (1) human hepatic cells exposed to kresoxym-methyl (7 µM) present an alteration in their mitochondrial function; (2) the combined exposure to kresoxym-methyl and boscalid alters the mitochondrial function of human hepatocytes at concentrations that were non-toxic when used individually. A previous screening on a panel of mitochondrial targeting agrochemicals suggested an induced alteration in oxygen consumption by human hepatocytes exposed to kresoxym-methyl (10 µM), as measured by the Seahorse XF technology [20]. Here, using EPR spectroscopy, we confirmed this alteration in OCR after kresoxym-methyl exposure (for concentrations equal or higher than 7 µM). In addition, we demonstrated that the alteration of OCR in hepatocytes led to a boost in mitochondrial superoxide radical anion production. As a consequence, defenses against oxidative stress were depleted, energy stores were altered and viability was decreased (Figure 1). These results are in the same line than our previous observations on pyraclostrobin, another strobilurin [21]. In addition, it was interesting to analyze the effect of a combined exposure to kresoxym-methyl together with boscalid. Boscalid belongs to the class of SDHIs for which toxicity concerns for human cells have been raised in the recent literature [31–33]. We observed a potentiating toxic effect (often called toxic cocktail effect) for the association kresoxym-methyl/boscalid (Figure 2), with an alteration of mitochondrial function at doses that did not present toxicity when used as single agent. Due to the scarcity of biomonitoring studies [34], there is a large uncertainty regarding the real blood levels concentrations that can be achieved after incidental exposure. Despite this lack of data, the concentrations at which we observed a mitochondrial dysfunction (when used alone or in combination) are relevant as they are in the range of concentrations that could be achieved after a single exposure at levels closed to the maximal tolerated exposure (ADI or AOEL) defined by authorities. Putting the results from our previous studies on boscalid [32], on pyraclostrobin [21], on their combination [21] and from the present study on kresoxym-methyl and its combination with boscalid, it becomes clear that agrochemicals targeting the ETC of fungi can also induce detrimental effects on the mitochondrial function of human cells. These observations could lead to a reconsideration of the balance benefice/risk and adaptation of regulation regarding the maximal tolerated levels of exposures. This seems particularly true for cocktails of fungicides. As discussed in our previous paper on the effects of pyraclostrobin, when used alone or in combination with boscalid [21], there is an urgent need for further research to assess the effect of prolonged or repeated exposure to fungicides, not only on hepatocytes, but also on cells more likely to be exposed (i.e. from the lungs, skin, and kidneys) or cells particularly sensitive to mitochondrial dysfunction (i.e. neurons).

**Figure 8.**
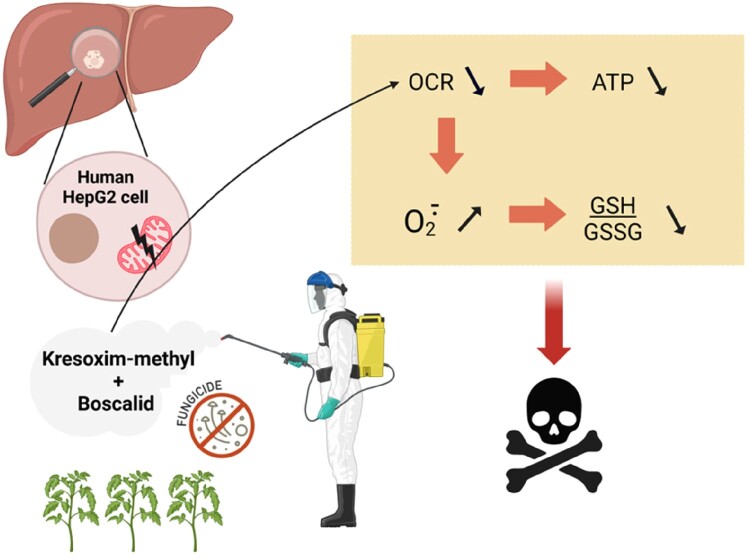
Summary of the main results from this study. Kresoxym-methyl, a strobilurin, and its mixture with boscalid, a SDHI, alter the mitochondrial function in human HepG2 cells: the OCR was decreased, the level of mitochondrial radical superoxide anion was increased, the ATP level was reduced, the ratio reduced/oxidized glutathione was decreased, with a consequent decrease in cell viability.

In conclusion, this study showed that the exposure to kresoxym-methyl, a fungicide from the class of strobilurins, alters the mitochondrial function in human HepG2 cells (Figure 8): the OCR was decreased, the level of mitochondrial superoxide radical anion was increased, the ATP level was reduced, the ratio reduced/oxidized glutathione was decreased, with a consequent decrease in cell viability. In addition, we observed an exacerbation of the toxicity when kresoxym-methyl was associated with boscalid, a SDHI.

## Supplementary Material

Supplementary_Figure_1.docx

## Data Availability

Rough data are available on request
