# Supplementary material for: Mitochondrial dysfunction induced in human hepatic HepG2 cells exposed to the fungicide kresoxim-methyl and to a mixture kresoxim-methyl/boscalid
Source: Redox Rep. 2024 Nov 14;29(1):2424677. doi: 10.1080/13510002.2024.2424677 (PMC11565682; doi:10.1080/13510002.2024.2424677)
Supplement: Supplementary_Figure_1.docx [file YRER_A_2424677_SM1731.docx]

Mitochondrial dysfunction induced in human hepatic HepG2 cells exposed to the fungicide kresoxim-methyl and to a mixture kresoxim-methyl/boscalid

Yasmine Vandensande^#^, Mélina Carbone^#^, Barbara Mathieu, and Bernard Gallez*

#: These authors contributed equally to this work

Biomedical Magnetic Resonance Research Group, Louvain Drug Research Institute, UCLouvain, Brussels, Belgium

**Supplementary Figure 1**

**
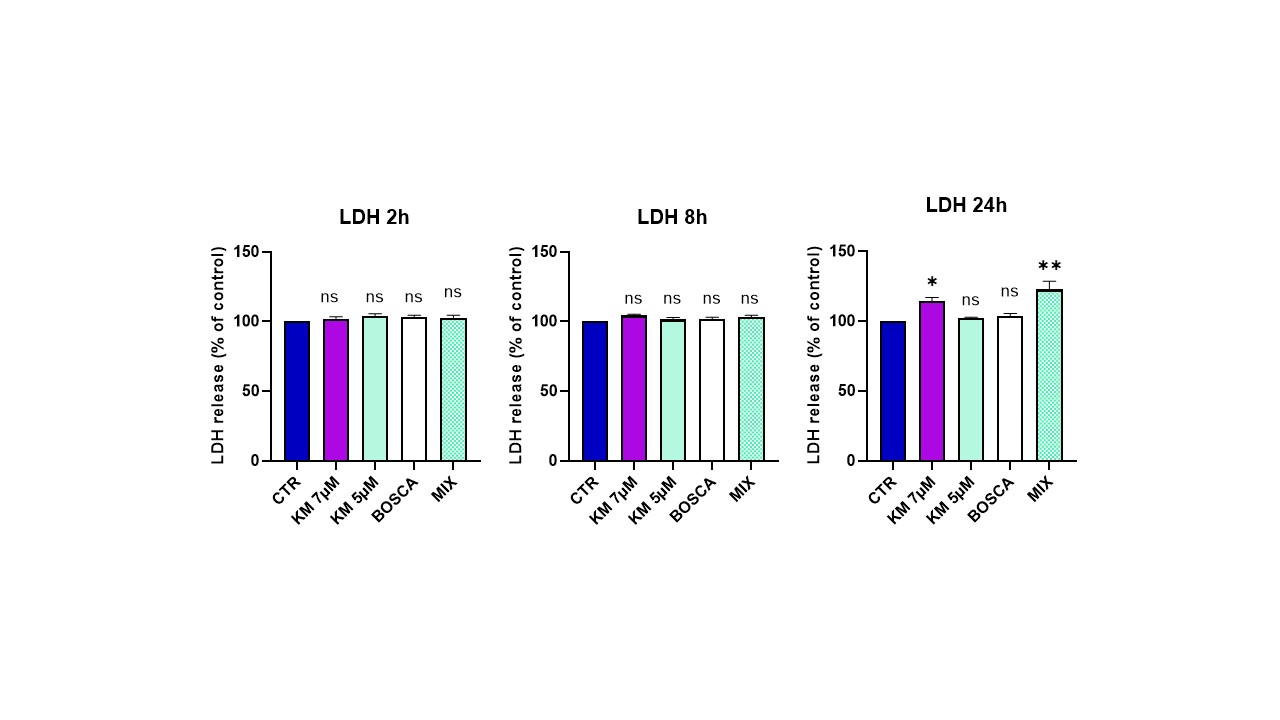
**

Effect of time exposure on cytotoxicity assessed by the LDH assay, N = 4, Anova One-way, Dunnett’s multiple comparison test, ; CTR = control, KM 7 µM = kresoxim-methyl 7 µM, KM 5 µM = kresoxim-methyl 5µM, BOSCA = boscalid 0.5 µM, MIX = kresoxim-methyl 5 μM/boscalid 0.5 µM mixture. Bars represent mean ± SEM. (*): p < 0.05, (**): p < 0.01, ns: not significant. p values are compared to control.
